# 

**Published:** 1907-04-15

**Authors:** 


### ANNOUNCEMENTS.

MICHIGAN STATE DENTAL ASSOCIATION: The annual meeting of this society will be held June 4th and 5th 1907, at Saginaw. The Board of Directors met in regular annual session in Detroit, March 26th and completed arrangements for a good program. The Association should have met at Saginaw last year, but the place of meeting was changed to Detroit in order that the 50th anniversary of the associations organization could be celebrated in the place where it was organized. There never was a time when the dentists of Michigan were so much interested in dental society work, and the prospects for a good program are very promising. The dentists of Saginaw will do all they can to make the welcome a cordial one.

MISSOURI STATE DENTAL ASSOCIATION: The annual meeting of this society will be held in Kansas City, June 4th, 5th and 6th 1907. A most interesting and profitable meeting is expected, and a royal welcome is assured. All ethical members of the profession are cordially invited to attend the meetings. F. G. Worthley, Pres. Kansas City, N. P. Dameron, Corresponding Secretary, St Louis.

LAKE ERIE DENTAL ASSOCIATION: This convention will meet at Cambridge Springs, Pa., on the 21st, 22nd and 23rd days of May 1907. This convention is always an attractive one for visitors, as the programes are always good and the social life and beautiful place of meeting assure a good attendance and a royal good time. Ethical dentists and their friends are cordially invited.

DETROIT DENTAL COLLEGE CLINIC AND BANQUET: On the 30th of May the commencement exercises will be held; and in connection with it a clinic and banquet will be held by the Alumni. The profession is cordially invited to participate. C. J. O'Reilly, Sec'y.

KENTUCKY DENTAL ASSOCIATION: Will hold its annual meeting May 20th, 21st and 22nd 1907, in Louisville. The profession is invited to attend, W. M. Randall, Sec'y, Louisville, Ky.

NEBRASKA EXAMINERS: Will meet in Lincoln, Neb., May 29th, 30th and 31st 1907. Applicants for examination must have their credentials in the hands of the secretary five days before the date. Dr. C. F. Ladd, Sec'y, Lincoln, Neb.

THE ARKANSAS STATE BOARD OF EXAMINERS: Will meet May 27th and 28th 1907, at Eureka Springs. The State Dental tal Association meets at the same place immediately following.

INDIANA STATE DENTAL ASSOCIATION: Will meet June 11th, 12th and 13th 1907, in Indianapolis. A cordial invitation is extended to the profession to attend, C. D. Lucas, Sec'y, Indianapolis, Ind.

TENNESSEE DENTAL ASSOCIATION: This societywill meet July 9th, 10th and 11th 1907, in Knoxville. A cordial invitation is extended to all ethical practitioners. R. J. Mc Gavock, Sec'y, Columbia, Tenn.

